# Supplementary material for: Chronic Cannabigerol as an Effective Therapeutic for Cisplatin-Induced Neuropathic Pain
Source: Pharmaceuticals (Basel). 2023 Oct 11;16(10):1442. doi: 10.3390/ph16101442 (PMC10610438; doi:10.3390/ph16101442)

Supplementary Material – Nachnani et al.

Table S1: TaqMan Gene Expression IDs for genes tested

Gene	Gene Name	TaqMan GEA ID
ActB	Beta-Actin	Mm02619580_g1
CNR1	Cannabinoid Receptor 1	Mm01212171_s1
CNR2	Cannabinoid Receptor 2	Mm02620087_s1
GPR55	G-protein coupled receptor 55	Mm02621622_s1
FAAH	Fatty Acid Amide Hydrolase	Mm00515684_m1
MGLL	Monoglyceride Lipase	Mm00449274_m1
ATF3	Activating Transcription Factor 3	Mm00476033_m1
TRPV1	Transient Receptor Potential Cation Channel Subfamily V Member 1	Mm01246300_m1
ADRA2A	Adrenergic Receptor 2A	Mm07295458_s1
ADRA2B	Adrenergic Receptor 2B	Mm00477390_s1
ADRA2C	Adrenergic Receptor 2C	Mm00431686_s1
DRD2	Dopamine Receptor D2	Mm00438545_m1
GFAP	Glial Fibrillary Acidic Protein	Mm01253033_m1
OPRM1	Mu Opioid Receptor 1	Mm01188089_m1
PParG	Peroxisome Proliferator Activated Receptor Gamma	Mm00440940_m1

Figure S1: qRT-PCR comparisons of gene expression in DRG (L3-L5) of male mice treated with 14 daily injections of CBG versus those treated with 14 daily injections of vehicle. Normalized to beta-actin. Statistical analyses performed by Mann-Whitney test, plotted mean with standard deviation. RQ; relative quotient

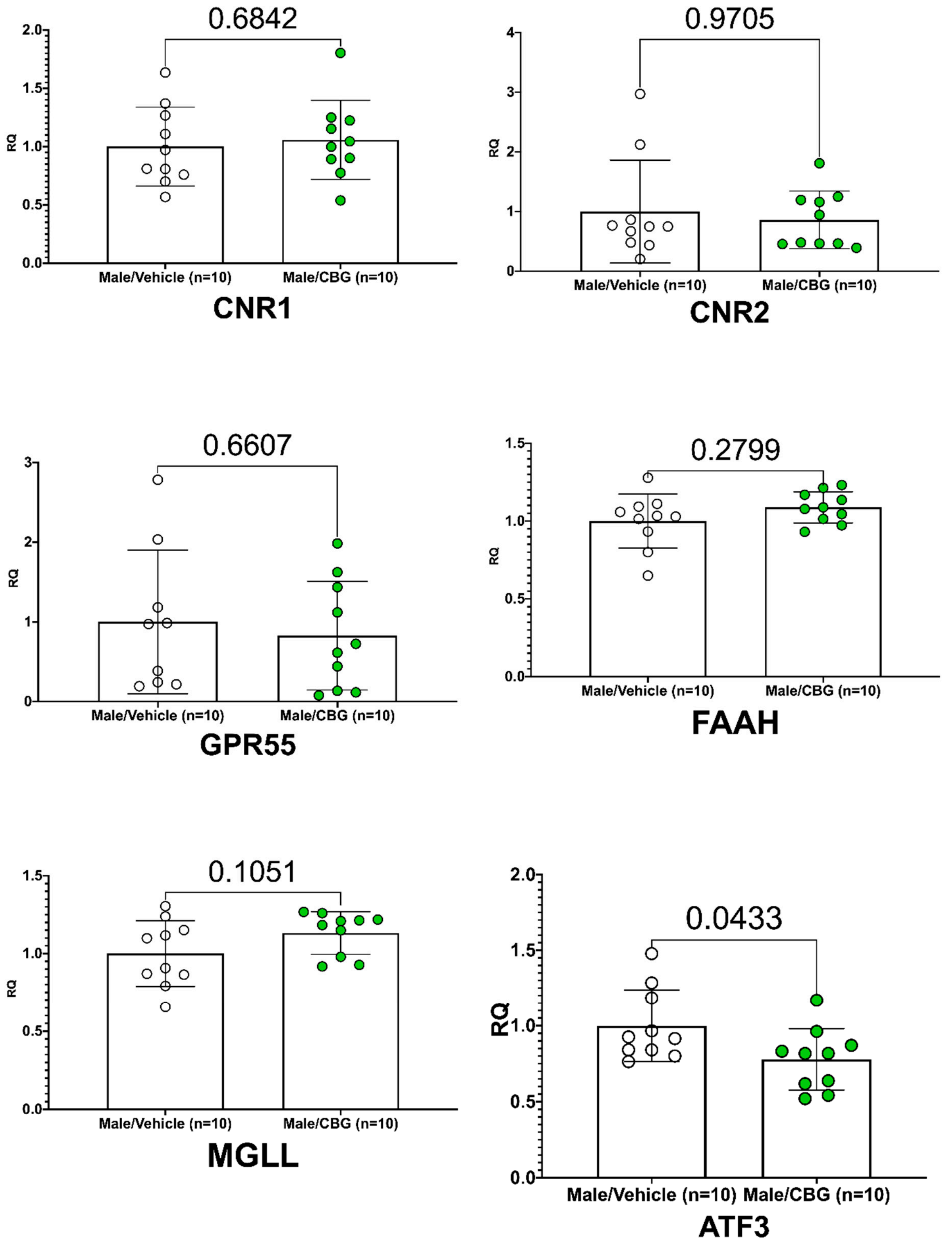

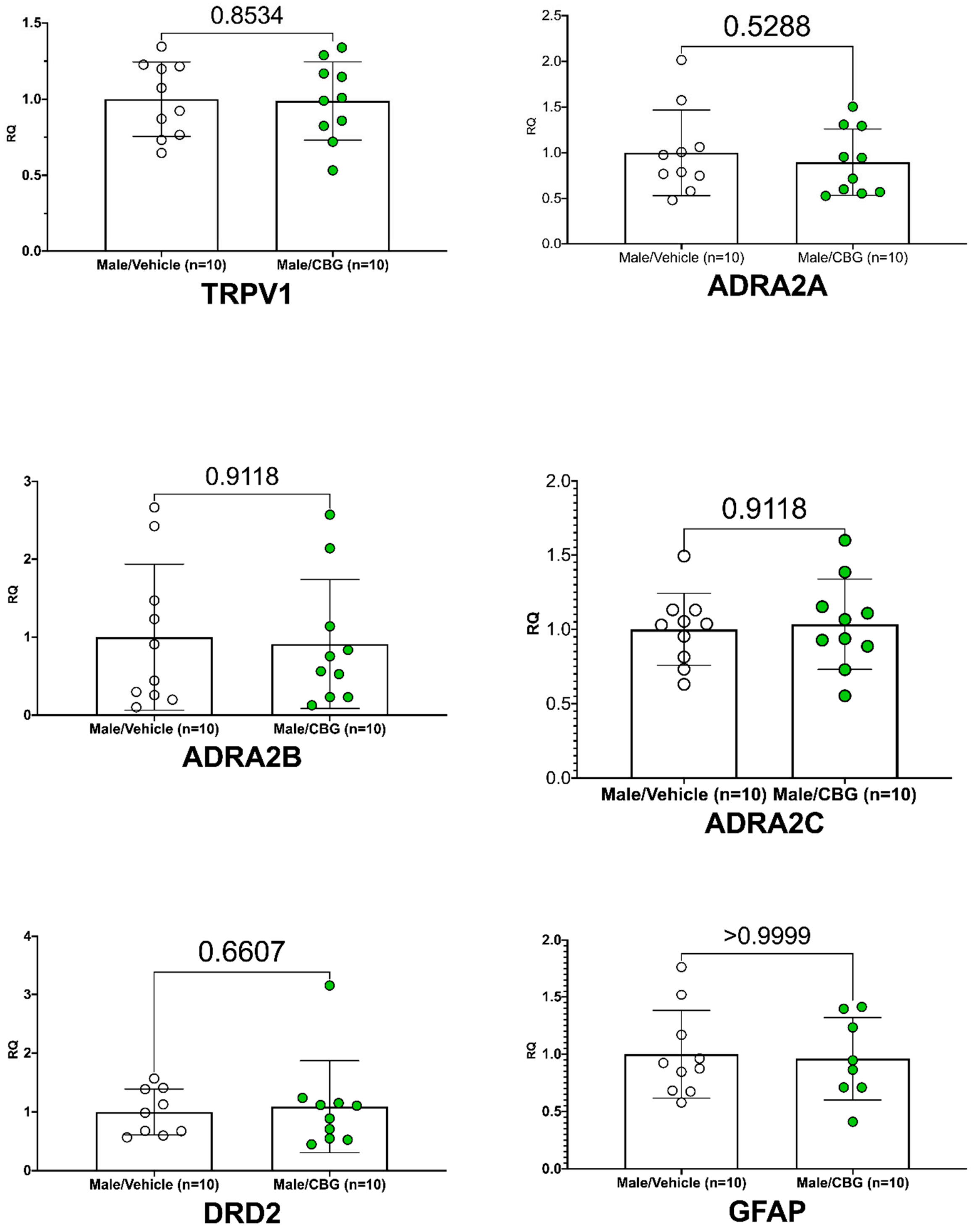

0.8534
RQ
Male/Vehicle (n=10)
Male/CBG (n=10)
TRPV1
0.5288
ADRA2A
0.9118
ADRA2B
ADRA2C
0.6607
DRD2
>0.9999
GFAP

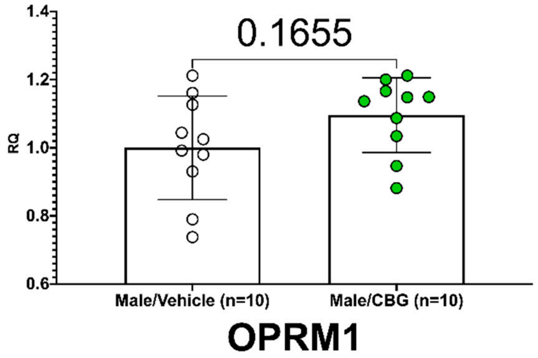
0.1655
RQ
1.4
1.2
1.0
0.8
0.6
Male/Vehicle (n=10)
Male/CBG (n=10)
OPRM1

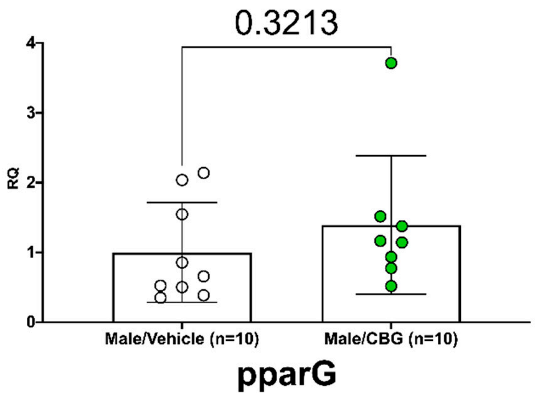
0.3213
RQ
4
3
2
1
0
Male/Vehicle (n=10)
Male/CBG (n=10)
pparG

Figure S2: qRT-PCR comparisons of gene expression in DRG (L3-L5) of female mice treated with 14 daily injections of CBG versus those treated with 14 daily injections of vehicle. Normalized to beta-actin. Statistical analyses performed by Mann-Whitney test, plotted mean with standard deviation. RQ; relative quotient

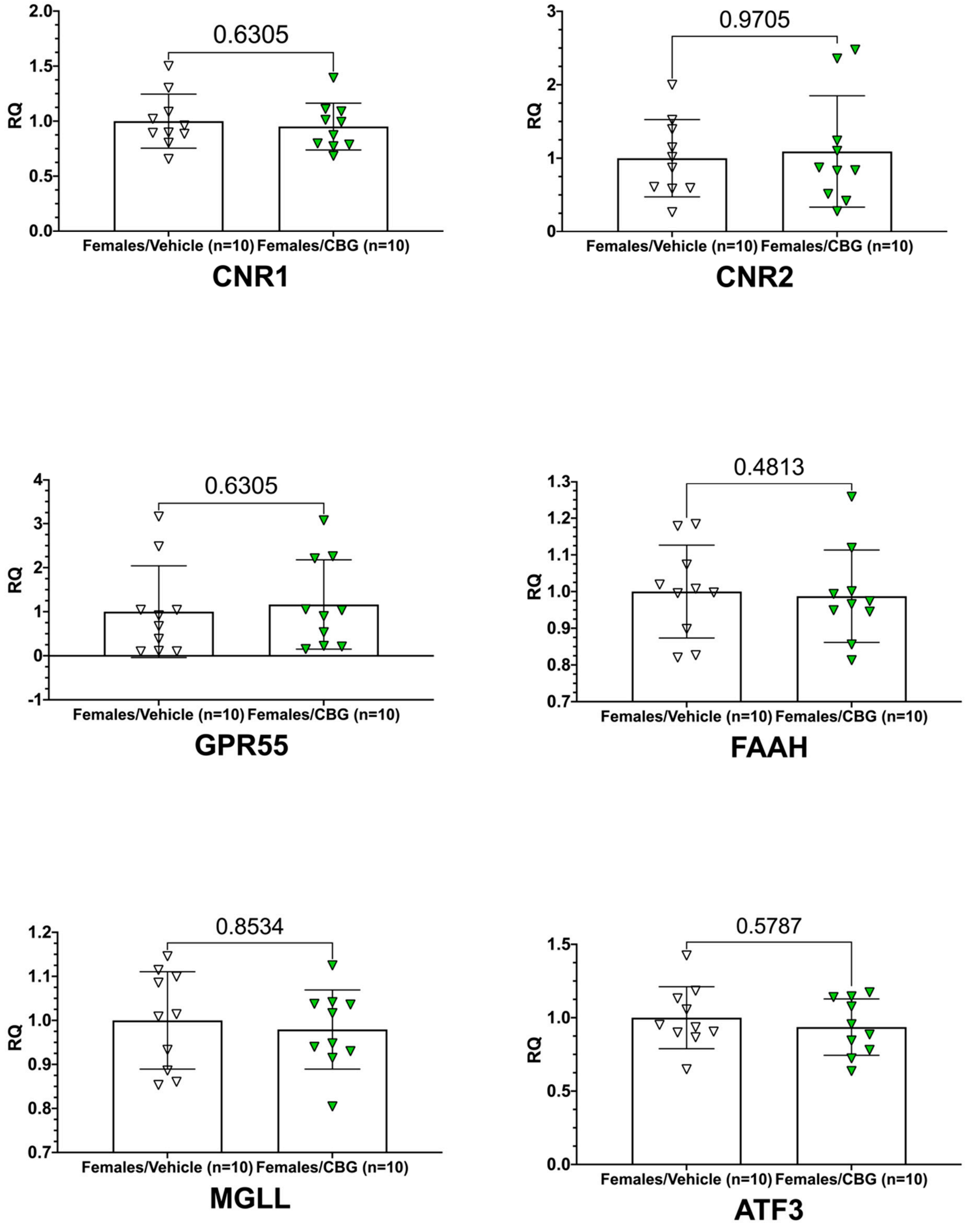

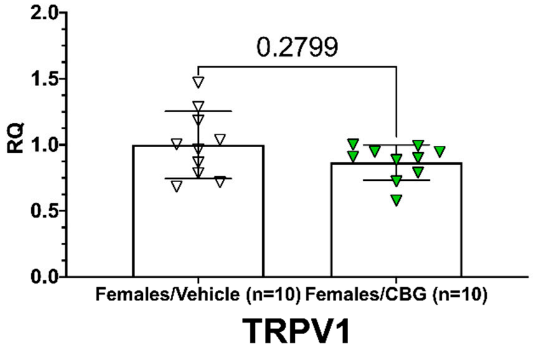
0.2799
RQ
Females/Vehicle (n=10)
Females/CBG (n=10)
TRPV1

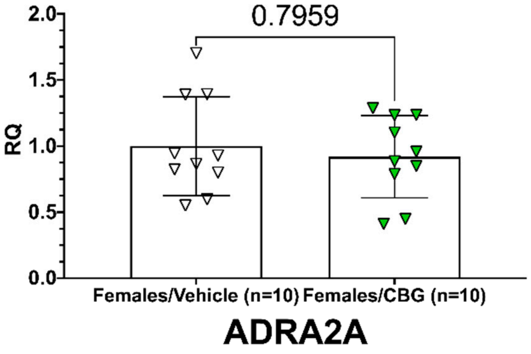
0.7959
RQ
Females/Vehicle (n=10)
Females/CBG (n=10)
ADRA2A

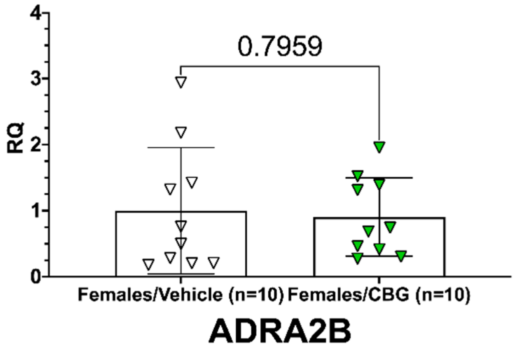
0.7959
RQ
Females/Vehicle (n=10)
Females/CBG (n=10)
ADRA2B

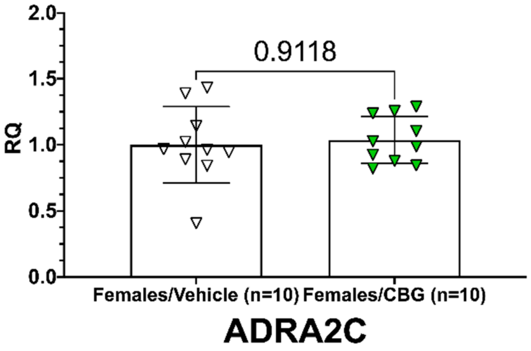
0.9118
RQ
Females/Vehicle (n=10)
Females/CBG (n=10)
ADRA2C

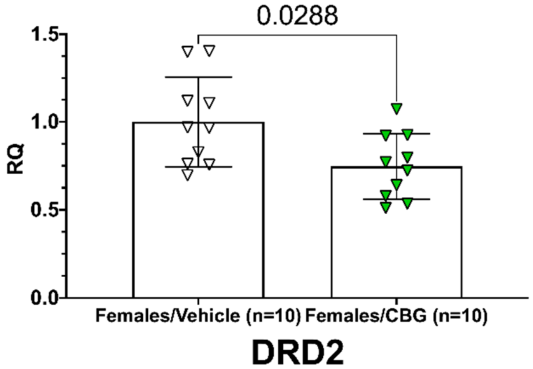
0.0288
RQ
Females/Vehicle (n=10)
Females/CBG (n=10)
DRD2

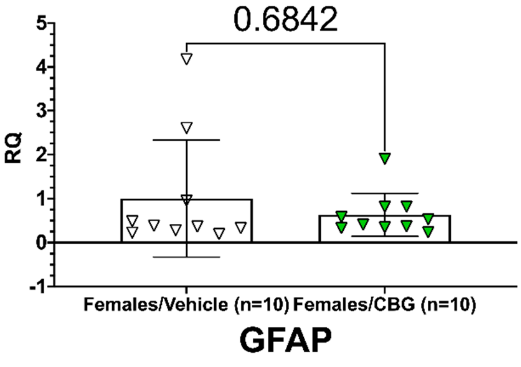
0.6842
RQ
Females/Vehicle (n=10)
Females/CBG (n=10)
GFAP

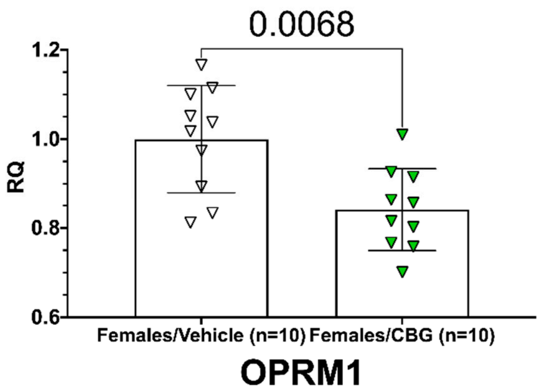
0.0068
1.2
1.0
0.8
0.6
RQ
Females/Vehicle (n=10)
Females/CBG (n=10)
OPRM1

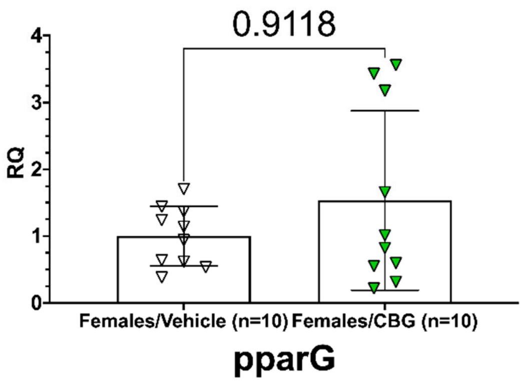
0.9118
4
3
2
1
0
RQ
Females/Vehicle (n=10)
Females/CBG (n=10)
pparG

Figure S3. Baseline-corrected graph of von Frey recordings of individual male and female baseline scores over time and their acute response to cannabigerol or vehicle. Data gathered one hour after injection. M/Vehicle = Males receiving vehicle. M/CBG = Males receiving cannabigerol. F/Vehicle = Females receiving vehicle. F/CBG = Females receiving cannabigerol. Mean with standard deviation.

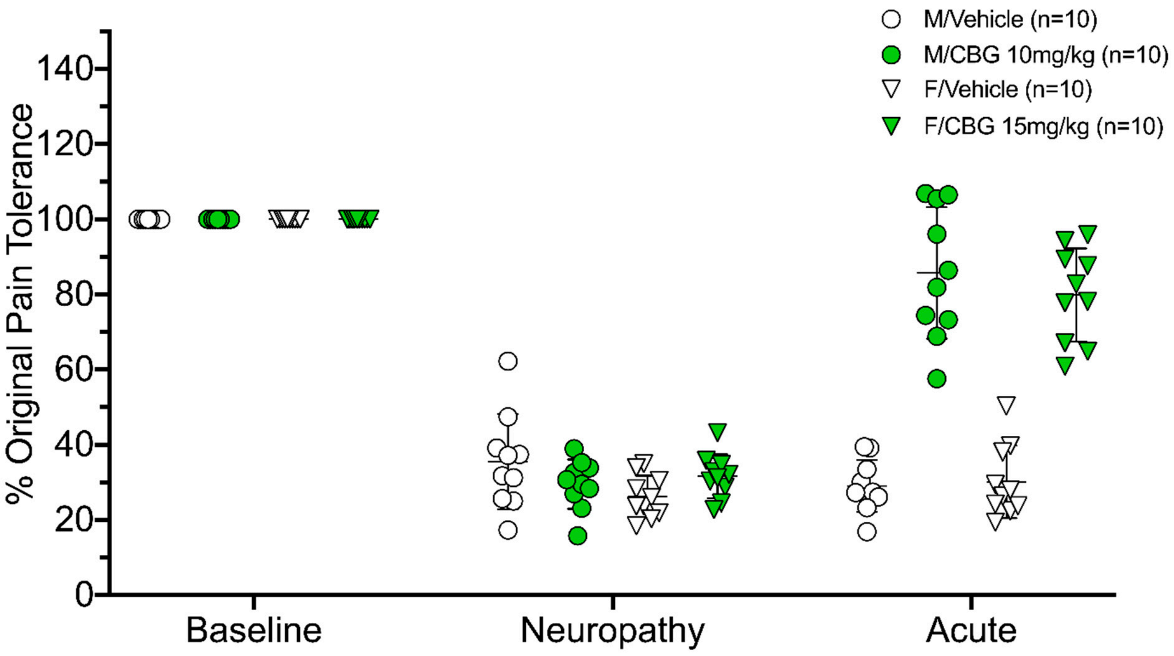

Figure S4. Baseline-corrected graph of von Frey recordings of individual male and female baseline scores over time and their chronic response to cannabigerol or vehicle. Data gathered twenty-four hours after seven and fourteen days of injections. M/Vehicle = Males receiving vehicle. M/CBG = Males receiving cannabigerol. F/Vehicle = Females receiving vehicle. F/CBG = Females receiving cannabigerol. Mean with standard deviation.

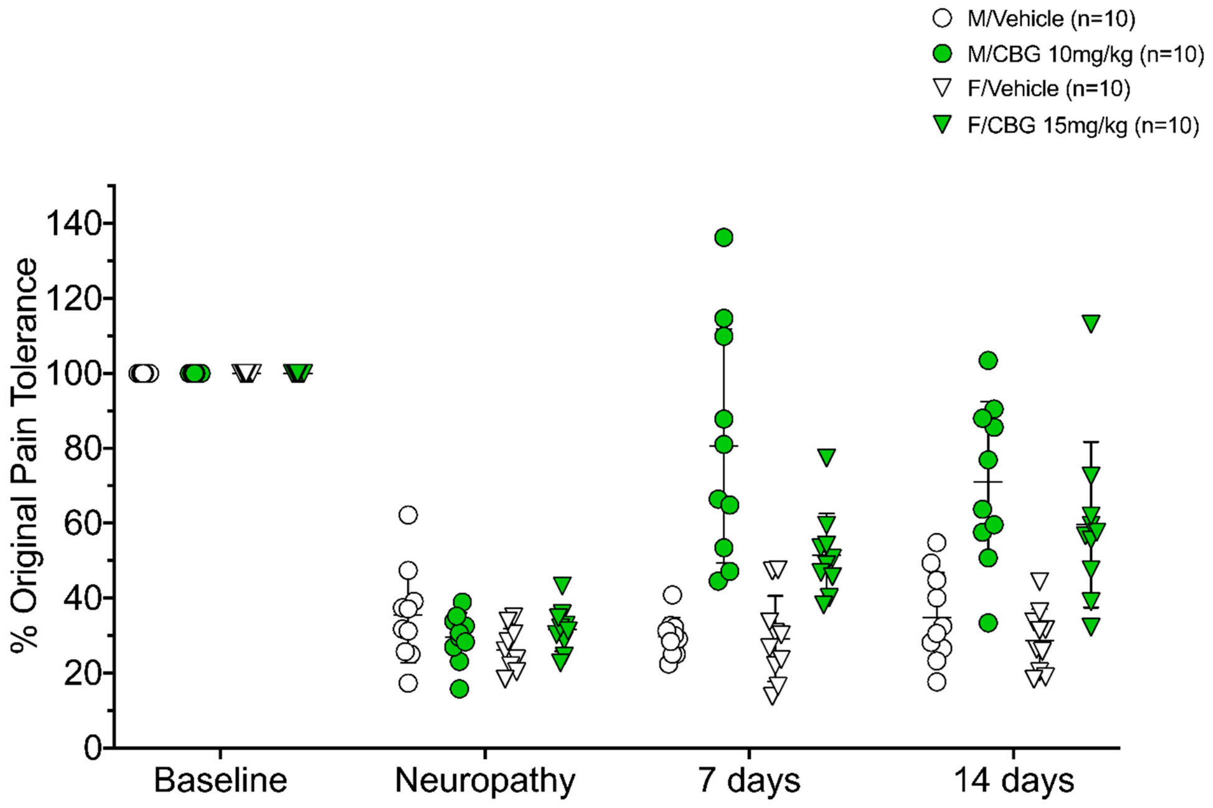

Figure S5. Weight over time in male (n=10 for vehicle and CBG) and female mice (n=10 for vehicle and CBG) receiving cannabigerol or vehicle treatments. Mice receiving CBG or vehicle did not experience any significant weight changes during treatment. CBG=Cannabigerol.

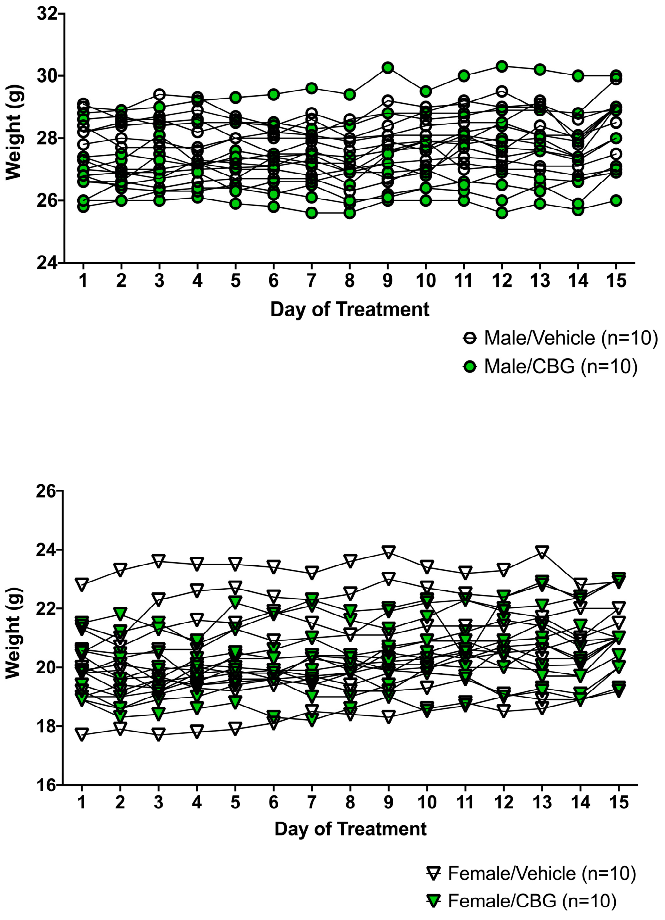

Supplement: Supplementary file 1 [file pharmaceuticals-16-01442-s001.zip › pharmaceuticals-2612394-supplementary.pdf]
